# Surface Modification of a Lignin-Derived Carbon-Supported Co-Based Metal/Oxide Nanostructure for Alkaline Water Splitting

**DOI:** 10.3390/molecules28155648

**Published:** 2023-07-26

**Authors:** Guoning Li, Faming Liu, Weiyang Ma, Hui Li, Shijie Li

**Affiliations:** School of Thermal Engineering, Shandong Jianzhu University, Jinan 250101, China; 2022030207@stu.sdjzu.edu.cn (F.L.); maweiyang27sdjzu@163.com (W.M.);

**Keywords:** lignin, biomass, water splitting, porous carbon, surface modification

## Abstract

Exploring low-cost and eco-friendly bifunctional electrocatalysts of the oxygen evolution reaction (OER) and hydrogen evolution reaction (HER) in alkaline electrolytes is still highly desired, and is crucial for water electrolysis and sustainable hydrogen generation. In this work, we report a facile pyrolysis–oxidation strategy to convert by-product lignin into bifunctional OER/HER electrocatalysts (Co/Co_3_O_4_-NPC-400) composed of Co/Co_3_O_4_ anchored on N-doped carbon with a surface of rich oxygen vacancies and oxygen-containing groups. The co-pyrolysis of lignin and NH_4_Cl can achieve a N-doped carbon matrix with a hierarchical pore structure, while the air-annealing process can induce the formation of oxygen-containing groups and oxygen vacancies. Owing to its surface properties, hierarchical pore structure and multiple active components, the constructed Co/Co_3_O_4_-NPC-400 possesses bifunctional catalytic activity and superior stability for OER/HER, especially for unexpected OER activity with a high current density of about 320 mA∙cm^−2^ at a potential of 1.8 V (vs. RHE). Water electrolysis using Co/Co_3_O_4_-NPC-400 as both the anode and the cathode needs a cell voltage of 1.95 and 2.5 V to attain about 10 and 400 mA∙cm^−2^ in 1 M KOH. This work not only provides a general strategy for the preparation of carbon-supported electrocatalysts for water splitting, but also opens up a new avenue for the utilization of lignin.

## 1. Introduction

With the massive consumption of fossil fuels in recent years, CO_2_ emission has become increasingly prominent, thus leading to global warming and climatic degeneration, which has spurred the development of sustainable energy. Hydrogen is a promising alternative to carbonaceous fuels due to its unique features of a substantial energy density and zero carbon content. However, 96% of global hydrogen comes from traditional production processes [1,2], such as coal gasification and natural gas reforming, which are inevitably accompanied by carbon dioxide emissions. Water splitting driven by electricity from renewable energies provides a preferred pathway to achieve green hydrogen, which can not only achieve carbon-free hydrogen production without generating greenhouse gases but also make full use of surplus renewable energy [3].

Water electrolysis involves two half-reactions, namely the oxygen evolution reaction (OER) on the anode and the hydrogen evolution reaction (HER) on the cathode. Admittedly, IrO_2_ and RuO_2_ have always been considered top-ranking OER catalysts, and platinum (Pt) exhibits the highest activity for HER, with nearly zero overpotential [4]. However, the natural rarity and high cost of these noble metals seriously impede their widespread application. Moreover, a bifunctional catalyst of HER/OER could simplify electrolyzer design and catalyst preparation, finally leading to a reduced cost of hydrogen production [5,6]. In this regard, transition metal-based catalysts, such as metal (Co, Ni, Fe, Mn, etc.) oxides, sulfides and phosphides, have been extensively studied as OER or HER catalysts for alkaline water splitting [7,8,9], owing to their universal stability and acceptable intrinsic activity in alkaline media. However, it is noted that the sluggish kinetics of OER caused by the four-electron transfer process generate a kinetic energy barrier, and water dissociation is also detrimental to HER kinetics [10,11], thus reducing the overall energy conversion efficiency of water electrolysis. Thus, it is still challenging to design effective electrocatalysts of OER or HER for alkaline water splitting.

Due to their low cost and eco-friendly characteristics, cobalt oxides have been recognized as promising electrocatalysts for OER, but they exhibit unsatisfactory HER activity. Recent studies have indicated that metallic Co has a lower H atom binding energy, indicating its potential HER activity [12,13]. However, these Co-based components still suffer from easy agglomeration, a low reactive surface area and poor electroconductivity, which reduce the active sites and hinder electron/ion transport [14,15]. Moreover, corrosion of these metals takes place under strong alkaline solution, which is detrimental to long-term stability [16,17]. To tackle the above puzzles, carbon materials, such as graphene, carbon nanotubes and activated carbon, can be employed as a matrix to support metal species due to their characteristics of structural diversity, tunable surface properties and decent chemical stability. Combining porous carbon with metal components can not only expedite mass transport but also make metal nanoparticles well dispersed, thereby increasing atom utilization and number of active sites. In addition, the carbon shell plays a role in protecting the metal core against corrosion [18]. As a result, the combination of metal, metal oxides and tailored carbon supports is a feasible strategy to construct Co-based hybrid catalysts for efficient overall water splitting.

Taking into account the features of rich carbon content, eco-friendliness and a neutral carbon cycle, biomass has received fairly extensive attention for the preparation of functional carbon materials. Among various biomass resources, lignin is the second most prevalent biological material in nature, after cellulose, accounting for 15~30% of lignocellulosic biomass [19]. As a by-product of the pulp and paper industry, technical lignin has an annual production of around 70 million tons. Unfortunately, only less than 5% of lignin is transformed into specialty chemicals, such as dispersants, surfactants and wood adhesives [20], while the rest is mostly used as low-value fuel or even abandoned as waste, which leads to the wasting of resources and to CO_2_ emission. Thus, converting lignin into high-added-value products for sustainable energy technologies is valuable work. Until now, major related studies have focused on the design of lignin-derived electrode materials for supercapacitors, lithium batteries and the oxygen reduction reaction, while lignin-derived electrocatalysts of OER or HER have been less reported. Based on the above, there is high demand for the preparation of low-cost and eco-friendly electrocatalysts for water splitting.

Inspired by the aforementioned ideas, we fabricated low-cost carbon-supported electrocatalysts of OER/HER, using lignin as a carbon source, through a two-step strategy containing pyrolysis followed by an air-annealing process. The influence of oxidation temperature on the properties and catalytic activity of the as-prepared catalysts was investigated. Due to the features of multiple Co-based active components, a hierarchical porous structure and a hydrophilic surface, the Co/Co_3_O_4_-NPC-400 catalyst displays bifunctional catalytic activities for OER/HER, especially enhanced OER performance at a high current density. The alkali electrolyzer using Co/Co_3_O_4_-NPC-400, as both the anode and the cathode, delivers current densities of 10 and 400 mA∙cm^−2^ at potentials of 1.95 and 2.5 V in 1 M KOH.

## 2. Results and Discussion

### 2.1. Material Characterization

**Figure 1:**
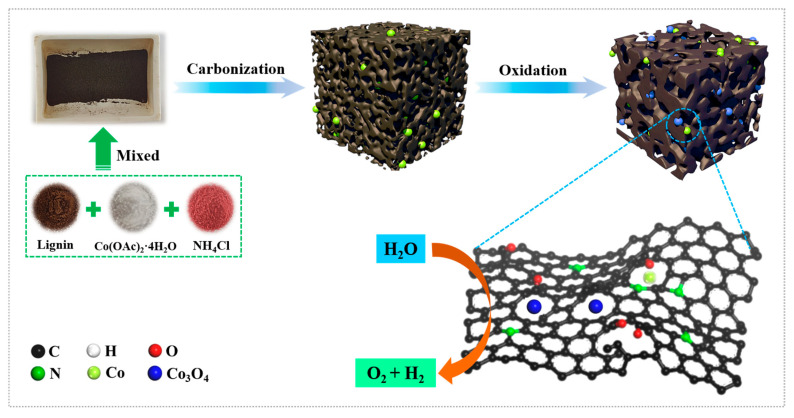
Schematic illustration of the synthetic process of Co/Co_3_O_4_-NPC.

**Figure 2:**
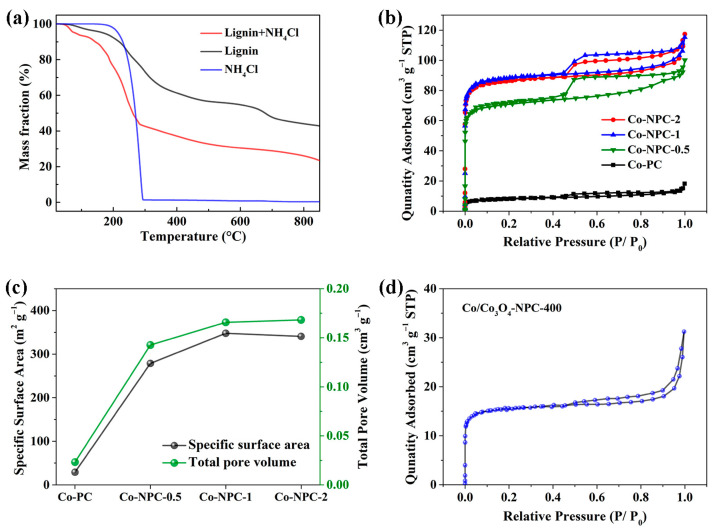
(**a**) TG curves of lignin, NH_4_Cl and their mixture. N_2_ adsorption–desorption isotherms (**b**) and comparison of BET specific surface area and total pore volume (**c**) of Co-PC and three Co-NPC-X samples. (**d**) N_2_ adsorption–desorption isotherm of Co/Co_3_O_4_-NPC-400.

Figure 1 displays a schematic diagram of the synthesis of Co-Co_3_O_4_ dual-active components embedded in N-doped porous carbon derived from lignin (Co/Co_3_O_4_-NPC). NH_4_Cl was applied as the nitrogen source and pore-forming agent due to the in situ released NH_3_ reacting with lignin during the pyrolysis process. To better understand the pyrolysis behavior of the precursor, a thermal analysis of NH_4_Cl, lignin and their mixture was conducted (Figure 2a). It can be found that the mixture can be roughly divided into three stages, among which the first (20~125 °C) and second (125~290 °C) weight losses are related to the evaporation of physically adsorbed water and the decomposition of NH_4_Cl into NH_3_ and HCl, respectively.

The effect of NH_4_Cl on the pore structure of lignin-derived carbon hybrid composites was studied via N_2_ adsorption and desorption. As shown in Figure 2b, the isotherms of all Co-NPC materials show a typical type-IV curve with a H4-hysteresis loop at P/P_0_ > 0.4 and sharp growth of N_2_ adsorption at low pressure, implying the presence of micropores, mesopores and macropores [21,22]. The BET specific surface area (SSA) and the pore volume were calculated and are summarized in Figure 2c. The SSA and pore volume of Co-NPC-1 are 347.75 m^2^∙g^−1^ and 0.1657 cm^3^∙g^−1^, respectively, which are much larger than those of Co-PC (29.09 m^2^∙g^−1^ and 0.0232 cm^3^∙g^−1^). Notably, we can see that the SSA of Co-NPC-1 is almost 12 times higher than that of Co-PC, further suggesting that NH_4_Cl can create a large quantity of pores in lignin-derived carbon materials and dramatically improve porosity. Furthermore, Co-NPC-2 has a similar SSA and pore volume to Co-NPC-1 but is larger than Co-NPC-0.5 (278.59 m^2^∙g^−1^and 0.1426 cm^3^∙g^−1^), also proving the influence of NH_4_Cl on the pore structure. These results illustrate that Co-NPC-1 prepared by using NH_4_Cl as a modifying agent has a hierarchical porous structure with a high surface area. In addition, Co/Co_3_O_4_-NPC-400 displays a similar N_2_ adsorption–desorption isotherm inherited from Co-NPC (Figure 2d), and the pore size distribution (Figure S1) calculated using the NLDFT model notably shows a relatively broad pore distribution, with the pores centered at 0.9~2.5 nm, 15~25 nm and 75~95 nm, suggesting that Co/Co_3_O_4_-NPC-400 has a hierarchical pore structure, which is beneficial for ion transfer and gaseous product removal from the pore structure of catalysts during the catalytic process. Moreover, Co/Co_3_O_4_-NPC-400 has a considerable SSA of 60.03 m^2^∙g^−1^ and a total pore volume of 0.0412 cm^3^∙g^−1^. Thus, it can be seen that Co/Co_3_O_4_-NPC-400 possesses a hierarchical pore structure with appreciable surface area, which can provide a decent active area to expose more approachable active sites, resulting in the enhancement of catalytic activity during the OER/HER process.

**Figure 3:**
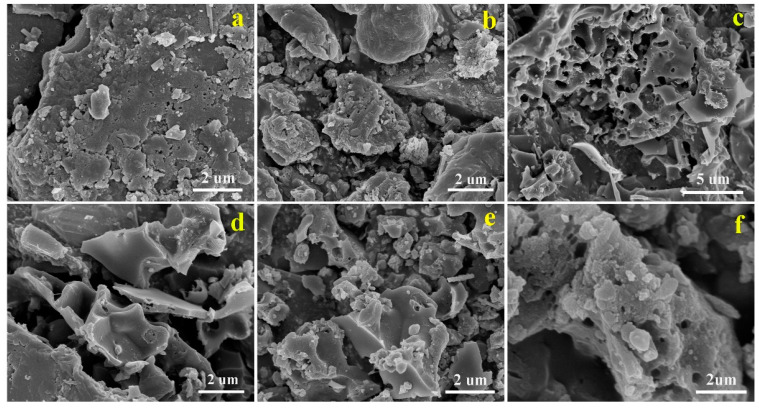
SEM images of Co-PC (**a**,**b**), Co-NPC (**c**,**d**) and Co/Co_3_O_4_-NPC-400 (**e**,**f**).

The micro-morphology of these synthesized materials was characterized via SEM. The lignin-derived carbon directly carbonized without NH_4_Cl modification shows a micro-sized block with an inferior pore structure (Figure 3a,b), while a honeycomb-like porous architecture composed of interconnected carbon nanosheets can be observed in Co-NPC-1 (Figure 3c,d). This above difference could be attributed to the addition of NH_4_Cl as the porogen. As depicted in Figure 3e,f, Co/Co_3_O_4_-NPC-400 inherited from Co-NPC-1 also presents a 3D interpenetrating framework with some sheet-like structure and a rough surface. Such a microstructure may effectively offer continuous pathways for electron and ion transfer during the electrochemical reaction, which is conducive to catalytic activity.

**Figure 4:**
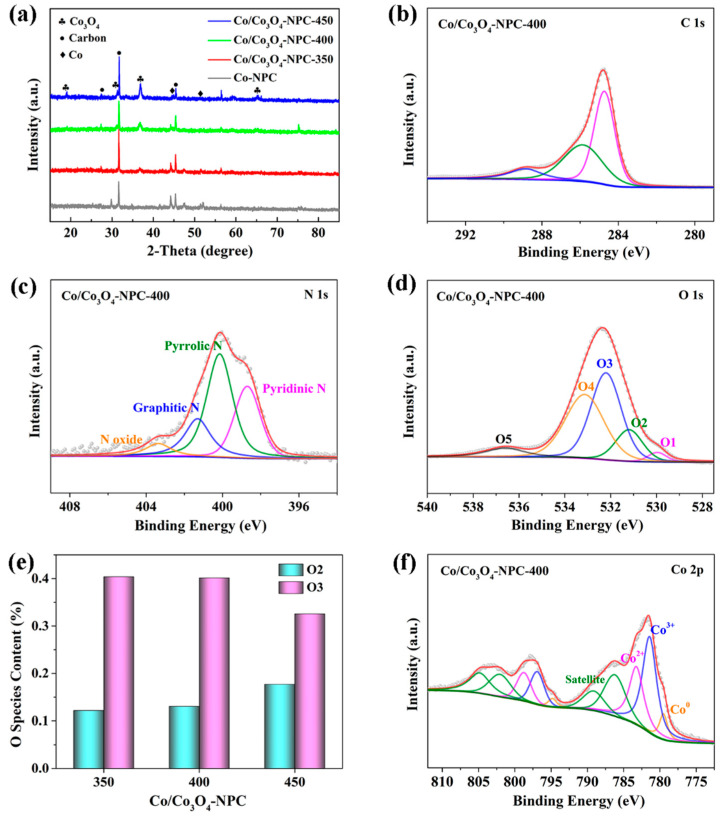
(**a**) XRD patterns of three Co/Co_3_O_4_-NPC materials and Co-NPC. (**b**) C 1s, (**c**) N 1s, (**d**) O 1s high-resolution XPS spectrum of Co/Co_3_O_4_-NPC-400. (**e**) Comparison of area ratios of O2 and O3. (**f**) Co 2p XPS spectrum of Co/Co_3_O_4_-NPC-400.

Figure 4a shows the XRD patterns of three Co/Co_3_O_4_-NPC materials and Co-NPC. The sharp diffraction peaks at 2θ = 27.4°, 31.7° and 45.4° can be attributed to crystalline carbon (JPCDS no. 46–0943), indicating the existence of crystalline carbon [23]. Moreover, the characteristic peaks located at 19.0°, 31.2°, 36.8° and 65.2° coincide well with the (111), (220), (311) and (440) lattice planes of Co_3_O_4_ (JPCDS no. 42-1467), respectively, and the peak of 44.2° corresponds to the (111) crystal facet of metallic Co (JCPDS no. 15-0806), thereby confirming the coexistence of Co and Co_3_O_4_ in three Co/Co_3_O_4_-NPC materials, especially Co/Co_3_O_4_-NPC-400. It also can be seen that Co species are present in the metallic phase in Co-NPC. Notably, with the oxidation temperature rising from 350 to 450 °C, the diffraction intensity of peaks assigned to the Co_3_O_4_ phase increases, while the peak of metallic Co significantly weakens, indicating that the ratio of metal to metal oxide could be controlled by the oxidation temperature, and an ideal temperature is crucial to the generation of the Co-Co_3_O_4_ heterostructure. The above analysis indicates that Co/Co_3_O_4_-NPC-400 has dominant Co_3_O_4_ and a small fraction of metallic Co, which is consistent with the following XPS results.

The surface states and elemental composition of the Co/Co_3_O_4_-NPC series were investigated via XPS analysis. As expected, the XPS survey spectra of these samples exhibit signals of C, N, O and Co (Figure S2). In the high-resolution spectra of C 1s (Figure 4b and Figure S3), there are three main peaks at 284.8, 286.2 and 288.8 eV, which are responsible for C–C, C–N and C=O (or C–O), respectively [21,24], indicating successful N-doping into the carbon skeleton and oxygen-containing groups on the surface. The high-resolution N 1s spectra (Figure 4c and Figure S4) can be classified into pyridinic N (~398.5 eV), pyrrolic N (~400 eV), graphitic N (401.2 eV) and N oxide (403–405 eV), respectively [25,26]. As previously reported, pyridinic N signifies the existence of metal-N bonds, and graphitic N infers the N-doping of the carbon skeleton, both of which are believed to be beneficial to the OER and HER processes [12,27,28]. Meanwhile, all the O 1s spectra (Figure 4d and Figure S5) present five main peaks at approximately 530, 531.2, 532, 533 and 536.5 eV, related to Co–O bonds (O1), hydroxyl species (O2), oxygen species bound to surface oxygen vacancies (O3), adsorbed water molecules (O4) and C–O bonds (O5) [29,30,31]. Calculated using the peak area (Figure 4e), Co/Co_3_O_4_-NPC-350 and Co/Co_3_O_4_-NPC-400 have higher O3 ratios than Co/Co_3_O_4_-NPC-450, suggesting more oxygen vacancies (O_vac_), as further evidenced by the following analysis of Co 2p. Furthermore, the higher the oxidation temperature, the larger the O2 ratio, indicating that hydroxyl groups are prone to forming at high oxidation temperatures.

As displayed in Figure 4f and Figure S6, the Co 2p spectra of Co/Co_3_O_4_-NPC-400 and Co/Co_3_O_4_-NPC-350 display two main peaks located at ~781.6 and ~797 eV with a spin energy separation of 15.4 eV, corresponding to the Co 2p_3/2_ and Co 2p_1/2_ spin-orbit peaks, respectively. These two Co 2p spectra can be deconvoluted into ten peaks, including Co^0^ (779.5 and 794.8 eV), Co^3+^ (~781.3 eV and ~796.8 eV), Co^2+^ (~783.1 eV and ~798.6 eV) and four accompanied shake-up satellites [32,33]. Likewise, Co/Co_3_O_4_-NPC-450 has two strong peaks of Co 2p_3/2_ (779.5 eV) and Co 2p_1/2_ (795.1 eV), and the energy interval of 15.6 eV is characteristic of the Co_3_O_4_ phase [34]. Two pairs of fitting peaks are attributed to Co^3+^ (779.7 and 794.9 eV) and Co^2+^ (781.8 and 797.1 eV), and no fitting peak of Co^0^ may be attributed to the limited detection depth (6–10 nm) of the XPS technique [35]. Based on the fitting peak of Co 2p_3/2_, Co/Co_3_O_4_-NPC-350 has a higher Co^0^ proportion (11.73%) than Co/Co_3_O_4_-NPC-400 (9.89%). More importantly, it has been generally accepted that the Co^2+^/Co^3+^ ratio is directly proportional to the number of oxygen vacancies [36]. Specifically, the Co^2+^/Co^3+^ ratios of Co/Co_3_O_4_-NPC-400 and Co/Co_3_O_4_-NPC-350 are 0.649 and 0.673, respectively, which are larger than that of Co/Co_3_O_4_-NPC-450 and the theoretical ratio of 1:2 in Co_3_O_4_, illustrating that the modest oxidation process could generate a lower oxidation state of Co and relatively more O_vac_ on the surface of catalysts, which is also confirmed by the O1s spectra. Due to the preferential catalytic activity of Co^2+^ and metallic Co for OER and HER, respectively [12,37], the mixed valence state of Co with a suitable proportion in Co/Co_3_O_4_-NPC can achieve highly effective Co-based electrocatalysts for overall water splitting. The XPS analysis confirms that the ratio of the valence state of Co species and the content of oxygen-containing groups in Co/Co_3_O_4_-NPC-T materials could be regulated by the oxidation temperature.

**Figure 5:**
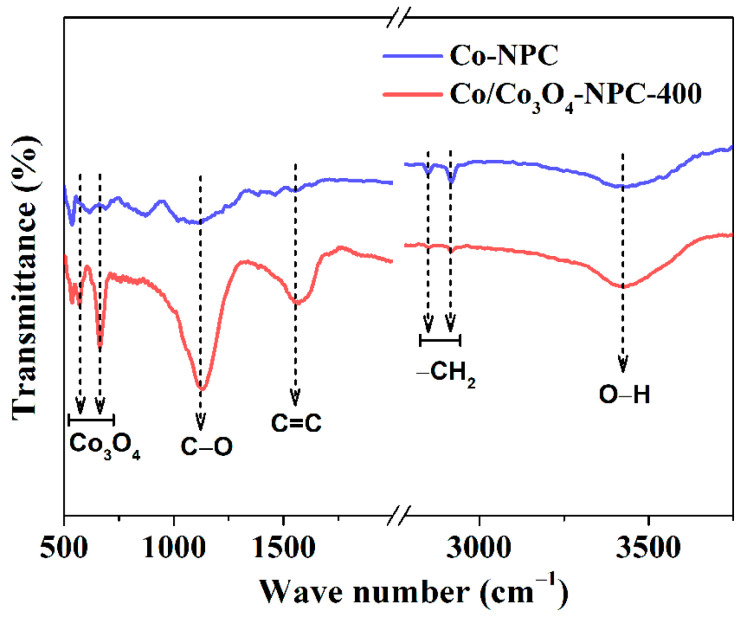
FTIR spectra of Co-NPC and Co/Co_3_O_4_-NPC-400.

FTIR spectra of Co-NPC and Co/Co_3_O_4_-NPC-400 were conducted to further explore the role of air-annealing treatment in the presence of various functional groups. As revealed by Figure 5, compared with Co-NPC, Co/Co_3_O_4_-NPC-400 has stronger peaks at 1131 and 3425 cm^−1^, which are attributed to the stretching vibration of aromatic C–O and hydroxyl groups (–OH) [38,39,40], demonstrating the formation of oxygen-containing groups on the carbon matrix after the oxidation process, which was also confirmed via XPS analysis. Moreover, the bands at 569 and 663 cm^−1^ appear in the spectrum of Co/Co_3_O_4_-NPC-400, which are ascribed to the vibrations of Co^3+^–O and Co^2+^–O in the spinel Co_3_O_4_, respectively [41]. In addition, two peaks at 2914 and 2850 cm^−1^ correspond to the –CH_2_ group, and the peak at 1566 cm^−1^ is caused by aromatic C=C stretching vibration [38]. The FTIR and XPS analyses reveal that Co/Co_3_O_4_-NPC-400 has abundant oxygen-containing functional groups, which would improve the hydrophilic property of the catalyst.

### 2.2. Electrocatalytic Performance of Co/Co_3_O_4_-NPC-400

**Figure 6:**
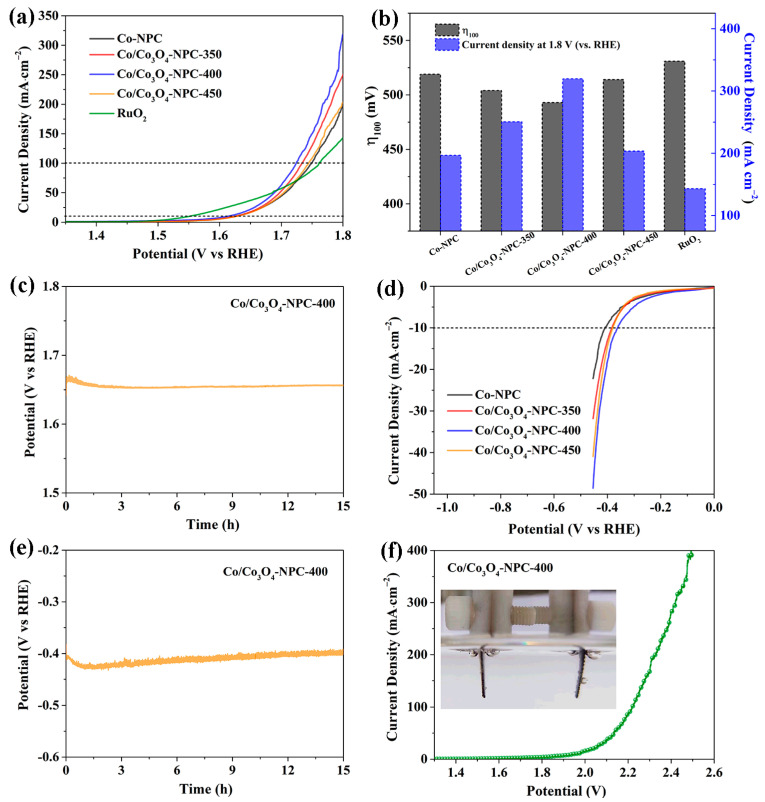
(**a**) OER polarization curves, (**b**) comparation of η_100_ and current density at 1.8 V (vs. RHE) of Co-NPC, Co/Co_3_O_4_-NPC and RuO_2_. (**c**) V-t curve of Co/Co_3_O_4_-NPC-400 for OER at 10 mA∙cm^−2^. (**d**) HER polarization curves of Co-NPC and Co/Co_3_O_4_-NPC. (**e**) V-t curve of Co/Co_3_O_4_-NPC-400 for HER at 10 mA∙cm^−2^. (**f**) LSV curve of the Co/Co_3_O_4_-NPC-400-based electrolyzer for overall water splitting.

Based on the above characterization analysis, it can be concluded that the dominant Co_3_O_4_ and a small fraction of metallic Co anchored on N-doped hierarchical porous carbon with a surface of rich O_vac_ and oxygen-containing groups (Co/Co_3_O_4_-NPC-400) was successfully synthesized. These features are beneficial to the OER and HER catalytic process. Currently, the electrocatalytic properties of OER or HER are always evaluated by directly applying as-prepared powder catalysts to a conductive substrate. Compared with metal-based substrates (e.g., Ni foam, Cu foam and Ti mesh), carbon cloth has the features of low cost, good thermal stability and low mass density [42]. As shown in Figure S7, carbon cloth has negligible OER and HER activity, which is helpful for analyzing the catalytic activity of the catalyst itself, when carbon cloth is used as a substrate to load the as-prepared powder catalysts. Thus, we explored the electrocatalytic activities of the Co/Co_3_O_4_-NPC series on carbon cloth for OER by conducting LSV measurements in 1.0 M KOH solution. For comparison, Co-NPC and commercial RuO_2_ were also assessed. As shown in Figure 6a, Co/Co_3_O_4_-NPC has a similar overpotential at a current density of 10 mA∙cm^−1^ (η_10_) of 383 mV with Co/Co_3_O_4_-NPC catalysts prepared at 350 and 450 °C, which is lower than Co-NPC (396 mV) but much higher than RuO_2_ (321 mV). More importantly, Co/Co_3_O_4_-NPC-400 exhibits much better catalytic activity than Co-NPC, RuO_2_ and the other two Co/Co_3_O_4_-NPC catalysts in the high-potential region, which requires an overpotential (η_100_) of 493 mV to achieve 100 mA∙cm^−2^ and can even acquire a high current density of 320 mA∙cm^−2^ at 1.8 V (vs. RHE). The specific data are presented in Figure 6b. To gain more insight into the catalytic activity, OER kinetics were further studied using Tafel plots, as shown in Figure S8a. The Tafel slope of Co/Co_3_O_4_-NPC-400 is 109.6 mV∙dec^−1^, which is obviously lower than RuO_2_ and the other as-prepared catalysts, inferring its more favorable OER kinetics and a more rapid OER rate at a high current density. These results indicate that Co/Co_3_O_4_-NPC-400 displays enhanced OER performance at a high current density. Moreover, the long-term stability of Co/Co_3_O_4_-NPC-400 for OER was evaluated using a chronopotentiometric (V-t) test. Figure 6c shows that the potential at the current density of 10 mA∙cm^−2^ was almost well maintained after 15 h, suggesting the superior OER stability of Co/Co_3_O_4_-NPC-400.

HER tests of the Co/Co_3_O_4_-NPC series and Co-NPC were also performed. As presented in Figure 6d, Co/Co_3_O_4_-NPC-400 possesses a rapid increase in the cathodic current along with lower onset potential and η_10_ (362 mV) as compared to the other three as-obtained catalysts. Also, Co/Co_3_O_4_-NPC-400 can attain a higher current density of 45 mA∙cm^−2^ at an overpotential of 450 mV, much larger than Co-NPC (21 mA∙cm^−2^), Co/Co_3_O_4_-NPC-350 (30 mA∙cm^−2^) and Co/Co_3_O_4_-NPC-450 (38 mA∙cm^−2^), further demonstrating better HER activity for Co/Co_3_O_4_-NPC prepared at 400 °C. Moreover, the Tafel slopes are shown in Figure S8b. Meanwhile, the V-t curve reveals that Co/Co_3_O_4_-NPC-400 shows excellent HER stability at 10 mA∙cm^−2^, with no obvious degradation of the potential for 15 h (Figure 6e). Based on the above, we can conclude that Co/Co_3_O_4_-NPC-400 is a viable electrocatalyst candidate for OER and HER in alkaline solutions. Hence, a two-electrode electrolyzer system was assembled using Co/Co_3_O_4_-NPC-400 as both the anode catalyst and cathode catalyst for overall water splitting in 1 M KOH solution. Figure 6f reveals that current densities of 10 and 400 mA∙cm^−2^ can be realized at potentials of 1.95 and 2.5 V, respectively, and the inset shows the generated bubbles (O_2_ and H_2_) on the electrode during the catalytic process.

**Figure 7:**
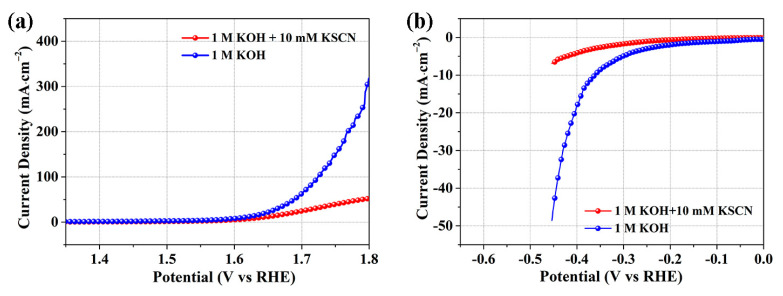
(**a**) OER and (**b**) HER polarization curves of Co/Co_3_O_4_-NPC-400 in 1 M KOH with or without 10 mM KSCN.

To disclose the metal-based active sites for OER and HER in Co/Co_3_O_4_-NPC-400, thiocyanate ion (SCN^−^)–poisoning experiments were performed in alkaline solution. It is well known that SCN^−^ ions can be strongly coordinated with Co ions to form highly stable complexes, thus leading to the poisoning of the Co-centered active sites [43,44]. As revealed in Figure 7a,b, Co/Co_3_O_4_-NPC-400 displays obvious activity loss for OER and HER after adding 10 mM KSCN in 1 M KOH solution, confirming that metal sites are crucial to the catalysis of OER and HER. Combined with the above analysis of XRD, XPS and BET characterizations, it can be seen that the bifunctional catalytic activity of Co/Co_3_O_4_-NPC-400 for OER and HER is predominantly contributed to by multiple metal-based active components, including Co-N_x_ active species, cobalt oxides with the favorable intrinsic OER activity and metallic Co with suitable Co–H binding energy [12,13], which could realize the integration of OER and HER active sites into a catalyst. Moreover, the rich surface O_vac_ can facilitate the pre-oxidation of Co^2+^ at relatively lower potential, which is beneficial for OER/HER activities [45,46]. Apart from the catalytic activity brought by the Co-based active sites, the hierarchical pore structure and hydrophilic surface from the N-doped carbon matrix could not only enable the active area to achieve more exposed active sites, but also promote electrolyte/catalyst contact and enhance mass transfer, especially gas product release, thus relieving the shielding effect of O_2_ or H_2_ bubbles under high-current-density conditions [47,48].

## 3. Experimental Section

### 3.1. Materials

Dealkaline lignin, ammonium chloride (NH_4_Cl) and isopropanol were obtained from Macklin Biochemical (Shanghai, China). Cobalt(II) acetate tetrahydrate (Co(OAc)_2_·4H_2_O) was purchased from Merck. All chemicals were used as received and without further purification. Carbon cloth was purchased from Taiwan Tanneng (Taiwan, China).

### 3.2. Synthesis of Co-Co_3_O_4_ Dual-Active Components Anchored on Lignin-Derived N-Doped Porous Carbon (Co/Co_3_O_4_-NPC)

Co-NPC was first prepared via a one-pot pyrolysis-activation process. In typical synthesis, 1 g of lignin powder, 2 mmol of Co(OAc)_2_·4H_2_O and a certain amount of NH_4_Cl were added to 20 mL of distilled water under stirring for over 5 h. Next, the mixture was dried at 80 °C overnight and further mixed via grinding in a mortar. The acquired powders were then pyrolyzed at 850 °C for 2 h (heating rate of 5 °C∙min^−1^) under a N_2_ atmosphere in a tube furnace. The pyrolysis products were named Co-NPC-X, where X is the mass of NH_4_Cl in g, and Co-NPC refers to Co-NPC-1 if not otherwise specified. For comparison, Co-PC was prepared without NH_4_Cl under the same conditions.

To obtain the Co/Co_3_O_4_ dual-active components, the Co-NPC powders were further treated via an air-annealing process at different temperatures (350, 400 and 450 °C) for 1 h. The resulting materials with different oxidation degrees were named Co/Co_3_O_4_-NPC-T, and T represents the oxidation temperature.

### 3.3. Material Characterization

The methodology and structure were characterized by the use of field emission scanning electron microscopy (FESEM, Apreo LoVac, FEI, Waltham, MA, USA) and a transmission electron microscope (TEM, JEOL JEM-2100, Tokyo, Japan). X-ray diffraction (XRD) measurements were conducted using a Rigaku SmartLab SE powder diffractometer. The X-ray photoelectron spectroscopy (XPS) spectra were recorded using a Thermo ESCALAB 250XI system (Thermo Fisher, Waltham, MA, USA). Fourier Transform Infrared (FTIR) was performed to reveal functional groups (Nicolet-6700, Nicolet, Waltham, MA, USA). The pore structure was studied using a gas sorption analyzer (ASAP-2460, Micromeritics, Norcross, GA, USA). The pyrolysis characteristics of the precursors were investigated using a Mettler Toledo TGA/DSC 3+ thermogravimetric analyzer (TGA).

### 3.4. Electrochemical Measurements

Electrochemical performance tests of OER and HER were performed on a CHI 760E electrochemical workstation (Shanghai Chenhua) with a three-electrode system in 1 M KOH solution. Hg/HgO was used as the reference electrode, and platinum for OER or a graphite electrode for HER was applied as the counter electrode. Carbon cloth (1 × 0.5 cm^2^) loaded with various catalysts was used as a working electrode, which was obtained based on the following steps: (i) A total of 5 mg of catalyst were evenly dispersed in 1 mL of a mixed solution containing deionized water isopropanol and Nafion solution via an ultrasound bath. (ii) The above-obtained catalyst ink was uniformly applied to carbon cloth and the loading mass was 0.5 mg cm^−2^. All the linear sweep voltammogram (LSV) curves were tested at a scan rate of 5 mV s^−1^ with IR corrected. All measured potentials were converted to a reversible hydrogen electrode (RHE) based on the following equation: *E*_RHE_ = *E*_Hg/HgO_ + 0.098 + 0.059 × pH.

## 4. Conclusions

To summarize, lignin-derived carbon-supported electrocatalysts have been successfully synthesized via a two-step pyrolysis-oxidation strategy, which is composed of the dominant Co_3_O_4_ and a small fraction of Co metal anchored on N-doped hierarchical porous carbon with a surface of rich O_vac_ and oxygen-containing groups. The results demonstrate that NH_4_Cl as a modifying agent can not only improve pore structure and increase surface area, but can also achieve N-doping for the carbon matrix during pyrolysis. Meanwhile, the valence state of Co and the formation of oxygen-containing groups could be affected by the oxidation temperature. Benefiting from multiple Co-based active sites, a hierarchical pore structure and surface hydrophilia, the resultant Co/Co_3_O_4_-NPC-400 exhibits bifunctional catalytic activities and superior stability, and requires a relatively low potential of 1.8 V (vs. RHE) to reach about 320 mA∙cm^−2^ for OER and an overpotential of 362 mV to achieve 10 mA∙cm^−2^ for HER. Co/Co_3_O_4_-NPC-400 displays especially unexpected OER performance, with a high current density of ~320 mA∙cm^−2^ at a relatively low potential of 1.8 V (vs. RHE). Moreover, the fabricated Co/Co_3_O_4_-NPC-400∥ Co/Co_3_O_4_-NPC-400 electrolyzer demands a cell voltage of 1.95 and 2.5 V for driving overall water splitting to reach about 10 and 400 mA∙cm^−2^, respectively. We believe that this facile strategy can be useful in designing low-cost and eco-friendly biomass-derived hybrid catalysts for application in electrochemical water splitting and other energy conversion reactions.

## Data Availability

Not applicable.

## Supplementary Materials

The following supporting information can be downloaded at: https://www.mdpi.com/article/10.3390/molecules28155648/s1, Figure S1: Pore size distribution of Co/Co_3_O_4_-NPC-400 calculated using NLDFT model; Figure S2: XPS survey spectra of the Co/Co_3_O_4_-NPC series; Figure S3: C 1s high-resolution XPS spectra of Co/Co_3_O_4_-NPC-350 and 450; Figure S4: N 1s high-resolution XPS spectra of Co/Co_3_O_4_-NPC-350 and 450; Figure S5: O 1s high-resolution XPS spectra of Co/Co_3_O_4_-NPC-350 and 450; Figure S6: Co 2p high-resolution XPS spectra of Co/Co_3_O_4_-NPC-350 and 450; Figure S7: (a) OER and (b) HER polarization curves of carbon cloth; Figure S8: The Tafel slopes of Co/Co_3_O_4_-NPC-400 for (a) OER and (b) HER.
