# Supplementary material for: Surface Modification of a Lignin-Derived Carbon-Supported Co-Based Metal/Oxide Nanostructure for Alkaline Water Splitting
Source: Molecules. 2023 Jul 26;28(15):5648. doi: 10.3390/molecules28155648 (PMC10419879; doi:10.3390/molecules28155648)
Supplement: Supplementary file 1 [file molecules-28-05648-s001.zip › molecules-2518517-supplementary.pdf]

Supporting Information

Article

Surface Modification of a Lignin-Derived Carbon-Supported Co-Based Metal/Oxide Nanostructure for Alkaline Water Splitting

Guoning Li *, Faming Liu, Weiyang Ma, Hui Li * and Shijie Li

School of Thermal Engineering, Shandong Jianzhu University, Jinan 250101, China; 2022030207@stu.sdjzu.edu.cn (F.L.); maweiyang27sdjzu@163.com (W.M.)
* Correspondence: liguoning20@sdjzu.edu.cn (G.L.); lihui2687459@163.com (H.L.)

Total number of pages: 4 (S1-S4)

Total number of figures: 8 (Figure S1-Figure S8)

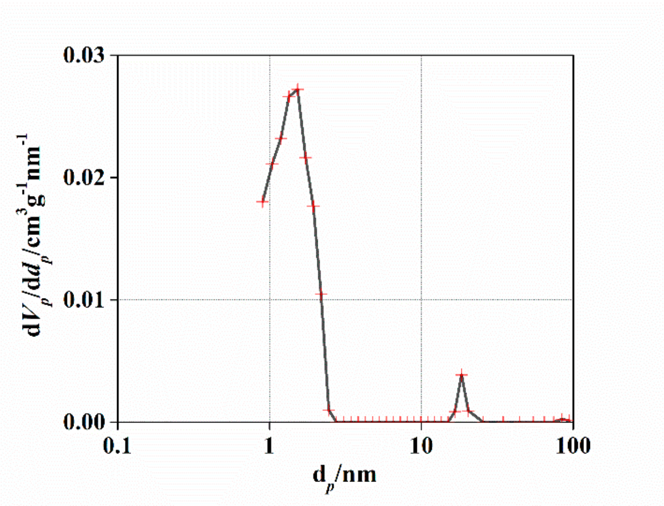

Figure S1. Pore size distribution of Co/Co_3O_4−NPC−400 calculated using NLDFT model.

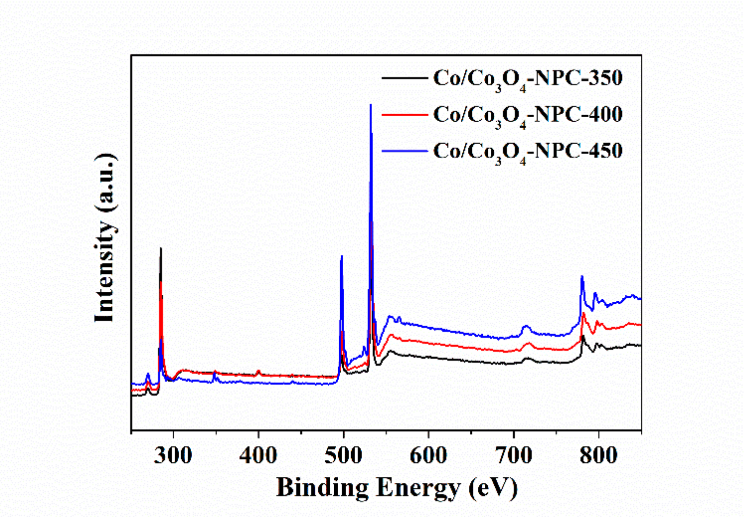

Figure S2. XPS survey spectra of the Co/Co_3O_4−NPC series.

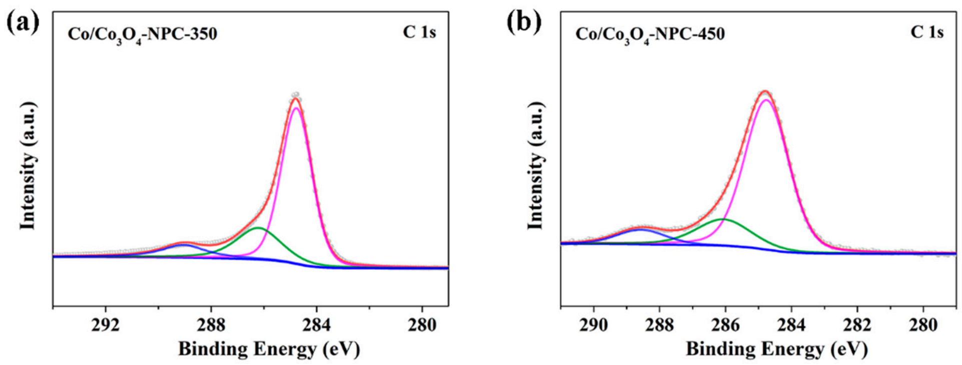

Figure S3. C 1s high-resolution XPS spectra of Co/Co_3O_4−NPC−350 and 450.

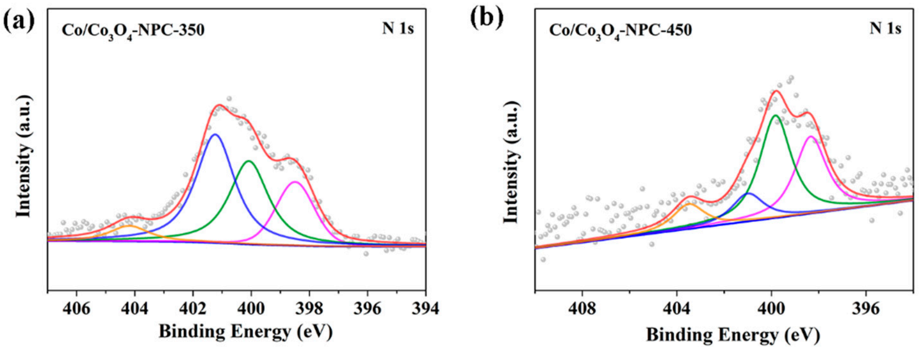

Figure S4. N 1s high-resolution XPS spectra of Co/Co_3O_4−NPC−350 and 450.

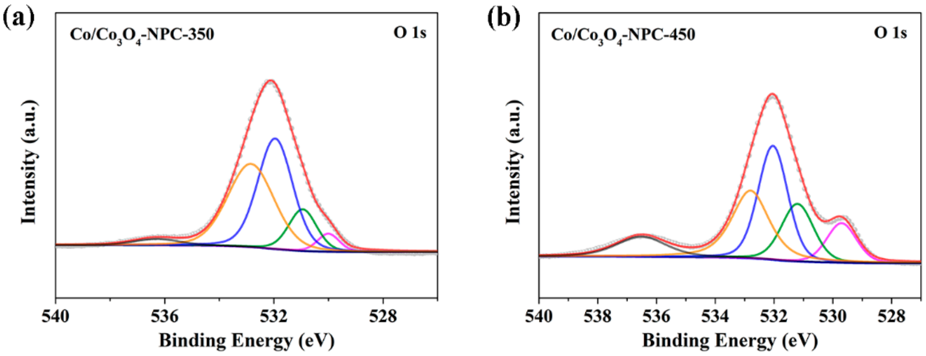

Figure S5. O 1s high-resolution XPS spectra of Co/Co_3O_4−NPC−350 and 450.

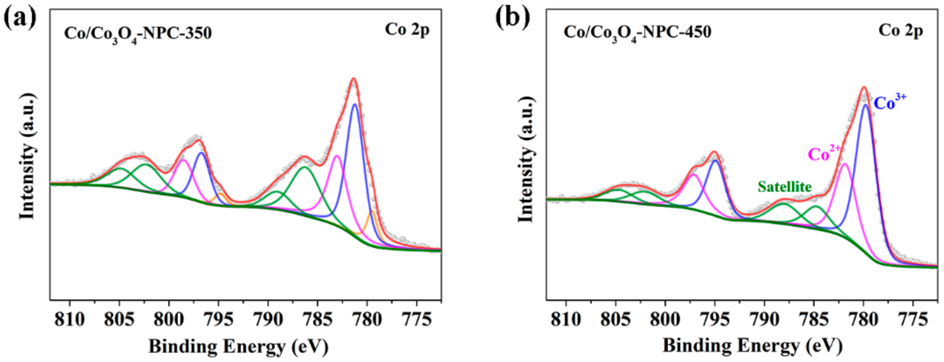

Figure S6. Co 2p high-resolution XPS spectra of Co/Co_3O_4−NPC−350 and 450.

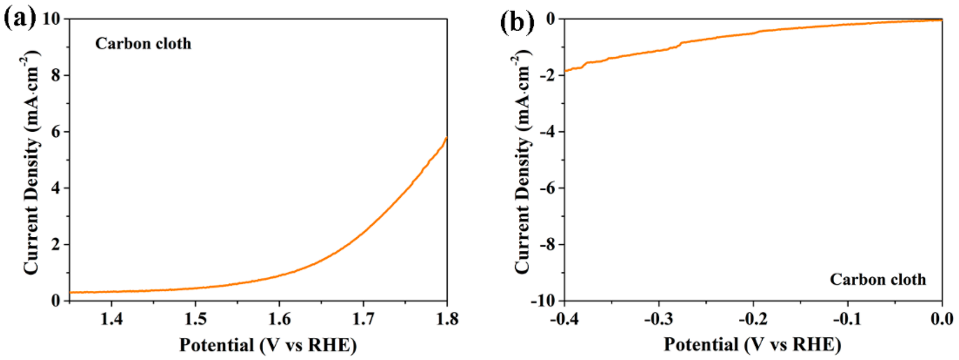

Figure S7. (a) OER and (b) HER polarization curves of carbon cloth.

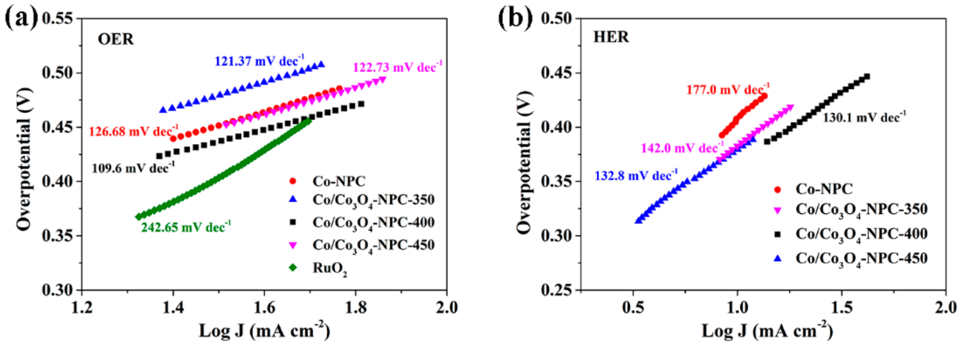

Figure S8. The Tafel slopes of Co/Co_3O_4−NPC−400 for (a) OER and (b) HER.
